# Daily Consumption of Lemon and Ginger Herbal Infusion Caused Tumor Regression and Activation of the Immune System in a Mouse Model of Breast Cancer

**DOI:** 10.3389/fnut.2022.829101

**Published:** 2022-04-13

**Authors:** Israa A. AL-ataby, Wamidh H. Talib

**Affiliations:** Department of Clinical Pharmacy and Therapeutic, Applied Science Private University, Amman, Jordan

**Keywords:** ginger, lemon, pinocytosis, apoptosis induction, angiogenesis, immunomodulatory activities

## Abstract

The Mediterranean diet includes the consumption of various fruits and vegetables. Lemon and ginger are highly popular in Mediterranean cuisine. The current study aims to evaluate both anticancer and immunomodulatory activities of lemon and ginger combination. The antiproliferative activities of the combination were tested against different cancer cell lines using 3-(4, 5-dimethylthiazol-2-yl)-2, 5-diphenyltetrazolium bromide assay. The degree of apoptosis induction and vascular endothelial growth factor expression were detected using ELISA. Balb/C mice were inoculated with the EMT6/P breast cancer cells and received combination water extract orally for 14 days. The effect of the water extract on splenocytes proliferation was measured using the mitogen proliferation assay. Macrophage function was evaluated using the nitro blue tetrazolium assay and pinocytosis was assessed using the neutral red method. Gas chromatography coupled to the tandem mass spectrometry was used to determine the composition of the combination. The lemon and ginger combination showed significant apoptosis induction and angiogenesis suppression effects. Fifty percent of the mice taking this combination did not develop tumors with a percentage of tumor reduction of 32.8%. This combination showed a potent effect in stimulating pinocytosis. Alpha-pinene and α-terpineol were detected in high percentages in the combination water extract. The lemon and ginger combination represents promising options to develop anticancer infusions for augmenting conventional anticancer therapies. Further testing is required to understand the exact molecular mechanisms of this combination.

## Introduction

Cancer is considered one of the widespread causes of death in the 21st century. Statistics reported 18.1 M new cancer cases and 9.6 M cancer deaths in 2018 (1). These numbers reflect the limited curative effect of conventional anticancer treatments (2). Many studies demonstrate that increased adherence to the Mediterranean dietary pattern is associated with health benefits (3). Herbal and alternative medicine is highly popular among cancer patients and it depends mainly on taking drinks and foods containing active ingredients (4). Food plays a vital role in cancer initiation and progression and some studies revealed a clear relationship between reduced cancer risk and dietary polyphenols (5). Many studies proved the effects of food in cancer treatment and prevention. A combination consisting of melatonin with a ketogenic diet showed promising results against drug-resistant breast cancer (6). While the combination of thymoquinone and piperine works synergistically against breast cancer by activating many mechanisms, namely, inhibition of blood vessels formation, stimulation of apoptosis, and modulating the immune response (7). Drinking of beverages, like tea and coffee or other products rich in antioxidants showed protective effects against neurodegenerative, cardiovascular, and cancer (8). Also, many natural products like curcumin had various biological influences, including anticancer activity. It acts as a regulator of p53 in breast cancer and is involved in regulating different molecular mechanisms (9).

Moreover, various dietary compounds exhibit anticancer effects by different mechanisms, namely, inhibition of tissue invasion and metastasis, modulation of the immune response, stimulating programmed cell death, and producing synergistic response with other anticancer agents (10–12). People with the greatest adherence to Mediterranean foods and drinks have more potential to avoid chronic diseases (13). Such a diet includes a high proportion of fruits and vegetables that provide a rich source of antioxidants. Lemon and ginger are important sources of active phytochemicals and both are popular ingredients in the Mediterranean diet (14, 15).

Lemon (*Citrus limonum*) relates to the Rutaceae family. The essential oil of lemon fruit peels is limonene (43.07%), a major bioactive component with a vital antifungal function (16). Citrus fruits are abundant in biologically effective phytochemicals that may protect against many cancer types. Previous studies confirmed the anticancer potential of citrus peels with potent activity reported for lemon peel (17). Furthermore, daily consumption of citrus fruits is connected with a diminished risk for gastric cancer (18). The ethyl acetate and petroleum ether extracts of citrus lemon have anticancer activity against various human cancer cell lines (19).

Ginger (*Zingiber officinale Roscoe*) relates to the Zingiberaceae family. In India and Nepal, ginger, lemon, and the black salt mixture were used widely to treat nausea (20). Gingerols and shogaols are essential components in ginger, both of them have biological activity, like anticancer, oxidative stress reduction, antimicrobial, anti-inflammatory, and antiallergic to multiple central nervous system activities (21).

Gas chromatography (GC) and GC−mass spectrometry (MS) of the essential oils from fresh and dried ginger rhizome revealed the presence of Zingiberene, which is considered a significant compound in both ginger oils (22). The methanol extract of ginger has an antiproliferation effect against human colorectal cancer cell lines (HCT116, SW480, and LoVo cells) (23). As well, an ethanol-water extract of dried ginger root presents antitumor activity against ovarian cancer cells (24).

Although lemon and ginger herbal infusion is highly popular globally, studies about the health benefits of this infusion are missing. In this study, a combination consisting of lemon and ginger water extract was tested for its anticancer and immunomodulatory activities *in vitro* and *in vivo* using a mouse model of breast cancer.

## Materials and Methods

### Plant Material and Extract Preparation

Dry herbs of the lemon and ginger combination used to prepare herbal infusions were purchased from the local market. These herbs are available in small bags ready for soaking in hot water. A water extract was prepared by soaking the herbal bag in hot water for 5 min and then drying the extract completely, using a rotary evaporator. The dried extract was weighed and stored at −20°C.

### Cell Line, Culture Conditions, and Mice

Six cell lines were used to examine the impact of the lemon and ginger combination water extract on their survival. Human epithelial breast cancer cell line (MCF-7), human breast adenocarcinoma cell line (MDA-MB-231), human colon carcinoma cell line (HCT-116), human adenocarcinoma alveolar basal epithelial cell line (A549), and EMT-6/P (mouse epithelial breast cancer) cell line were used in this study. MCF-7 cell line was cultured in the complete Roswell Park Memorial Institute (RPMI)-1640 medium. The MDA-MB231, HCT-116, and A549 cell lines were cultured in the complete Dulbecco’s modified eagle medium (DMEM) medium-high glucose. Mouse epithelial breast cancer cell line (EMT-6/P) was cultured in a minimal essential medium. Kidney epithelial cells from the African green monkey (Vero) were used as normal control and cultured in the complete DMEM medium. All the six cell lines were incubated at 37°C in a 5% CO_2_ and 95% humidity incubator. All media mentioned above were supplemented with 10% fetal calf serum, 1% L-glutamine, 0.1% gentamycin, and 1% penicillin-streptomycin solution.

Untreated cells (wells only contain cells plus tissue culture media) were used as the negative control. While cells treated with vincristine sulfate are considered as positive controls. Vincristine is a standard anticancer drug derived from the plant called *Catharanthus roseus*, it was classified as a plant alkaloid. Both of these controls were used to compare the activity of our extract (25).

Balb/C healthy female mice aged between 4 and 6 weeks and weighing between 23 and 25 g were utilized in this research. The mice were kept in separated cages with wooden shavings used as bedding. The animal room’s environmental parameters were 50–60% humidity, 25°C temperature, and continuous air ventilation. Animal care and use were conducted according to standard ethical guidelines and the Research and Ethical Committee approved all of the experimental protocols at the Faculty of Pharmacy in Applied Science Private University.

### Antiproliferative Assay

Each of the six-cell lines (actively growing) was collected by the trypsinization method and seeded into 96-well flat-bottom plates at a density of 13,000 cells/well for 24 h. At the end of the incubation period, cells were treated (in triplicate) with gradually increasing concentrations of lemon and ginger water extract (25–0.19 mg/ml). The extract was sterilized using 0.2-um syringe filters. Cells were kept with the extracts for 48 h; later, the cell survival was measured using 3-(4, 5-dimethylthiazol-2-yl)-2, 5-diphenyltetrazolium bromide (MTT) assay. A microplate reader (Biotek, Winooski, VT, United States) was used to detect the resulting change in color at 550 nm. The percentage survival was measured for treated cells and compared with untreated cells. Untreated cells were used as negative controls and cells treated with vincristine sulfate (0.05–0.00039 mg/ml) were used as positive controls (26).

### Determination of Vascular Endothelial Growth Factor Expression in MDA-MB231 Cells

The MDA-MB231 cells were seeded into three separated tissue culture flasks at the concentration of 150,000 cells per ml. After overnight incubation, the old media were discarded and cells were treated with one of the following treatments: lemon and ginger combination water extract (3.5 mg/ml), vincristine sulfate (0.025 mg/ml), as a positive control, and the third flask considered as a negative control [tissue culture media + 0.1% dimethyl sulfoxide (DMSO)]. The three flasks were placed in the incubator for 48 h. After that, the media of each flask were transferred into sterile tubes and vascular endothelial growth factor (VEGF) levels were measured using ab222510 Human VEGF Simple Step Elisa^®^ Kit version 1 catalog (27). A standard curve was obtained using the Human VEGF Simple Step ELISA Kit at various concentrations.

### Apoptosis Detection in MDA-MB231 Cells

MDA-MB231 cells were dispensed into three separated tissue culture flasks at a concentration of 150,000 cells/ml. After 24 h, cells were treated with one of the subsequent treatments.

Lemon and ginger combination water extract (3.5 mg/ml), vincristine sulfate (0.025 mg/ml) and the negative control (tissue culture media + 0.1% DMSO). Cells were incubated for 48 h with different treatments (27). Then the media of each flask (three flasks) were removed and the attached cells were harvested. Caspase-3 activity was measured using ab39401 Caspase-3 Assay Kit (Colorimetric). Fold-increase in caspase 3 activity was measured by comparing extract results with the negative control.

### Antitumor Activity on Experimental Animals

The duration of this study was 4 weeks, conducted on 20 healthy females Balb/C mice. Throughout the first 2 weeks, controlling the diet of 10 mice by giving them: a lemon and ginger combination of 14.3 mg/kg/day orally (gavage feeding). In the last 2 weeks, the same dose was administrated daily, but after inoculating (subcutaneously) each mouse with (100,000 cells/0.1 ml) EMT-6/P cell line (at day 14) (7).

The *in vivo* chosen dose was to mimic the way of preparation and concentration given to humans daily. The remaining untreated 10 mice were considered as a negative control group [inoculated with cancer at day 14 and treated with 0.1 ml phosphate-buffered saline (PBS) orally]. Tumors growth was monitored using a digital caliper, tumor sizes were measured and the volume of each tumor was calculated based on the following formula: (A × B^2^ × 0.5) (28). At the end of the 4th week, the mice were humanely killed and their tumors have been removed, weighed, and kept in 10% formalin.

### Preparation of Murine Splenocytes

A healthy Balb/C mouse was sacrificed and its spleen was removed aseptically. A tissue grinder was used to prepare a splenocytes suspension; mainly by passing the spleen through it. Cells were washed three times then re-suspended in 0.15 M NH_4_Cl solution to break down RBCs. After 10 min, the cells were repeatedly centrifuged and re-suspended in RPMI-1640 media for further use in other assays (29).

### Lymphocytes Proliferation Assay

3-(4, 5-Dimethylthiazol-2-yl)-2, 5-diphenyltetrazolium bromide (MTT) assay kit (Bioworld, Philadelphia, PA, United States) was used as the main function in this assay. Splenocytes suspension was prepared (5 × 10^6^ cell/ml) in RPMI-1640 and followed by seeding in a 96-well culture plate in the presence of 2 μg/ml Con A or 4 μg/ml lipopolysaccharide (LPS). To this, 100 μl of decreasing concentrations (25–3.125 mg/ml in RPMI-1640) of lemon and ginger combination water extract were added separately (in triplicates) followed by incubation for 48 h. Following the incubation, 10 μl MTT (5 mg/ml) solution were added to each well. The plate was coated with aluminum foil and then incubated for 4 h. One hundred μl DMSO was then added to each well to end the reaction and the absorbance was measured at 550 nm (29). Results were summarized as a percentage of survival (%) compared to the untreated cells (negative control). The same procedure was repeated but without Con A and LPS.

### Macrophage Isolation From Peritoneal Fluid

Peritoneal macrophages (PEM) were obtained from 5 Balb/C mice, which were previously injected with 3 ml of thioglycollate (intraperitoneal) 72 h before the experiment day. The mice were euthanized by cervical dislocation. The outer layer of the peritoneum is shear by forceps and scissors then pull it out quietly to discover the inner layer that lines the peritoneal cavity. Moreover, 5 ml ice-cold PBS was injected into the abdominal cavity. After gentle massaging to the peritoneum area (to expel and migrate cells into the PBS solution), the fluid was collected by inserting a 5 ml syringe into the peritoneum and start collecting the fluid, whereas, moving the tip of the needle gently to dodge hampering by the fat tissue or others. Later, it was placed in a centrifuge tube held on ice. If any visible blood contamination was detected in the fluid sample, then the sample should be removed (30).

The method was repeated five times and the fluids were pooled. After the centrifugation of the pooled fluid (3,000 RPM, 10 min, 4°C), each cell pellet was suspended in RPMI 1640 medium. The cells were counted and seeded at a concentration of 5 × 10^6^ cells/well in a 96-well microplate, then incubated to adhere for 3 h at 37°C in a 5% CO_2_ humidified incubator. After that, the non-adherent cells were washed away with a medium and the adherent cells were used in the different assays outlined below (29).

### *In vitro* Phagocytic Assay

The NBT (nitro blue tetrazolium) reduction assay was performed according to Rainard’s method (31). The Peritoneal macrophages (PEM) (5 × 10^6^ cells/well of a 96-well plate) were cultured with different concentrations of the lemon and ginger combination water extract (12.5–1.56 mg/ml) for 48 h at 37°C. After that, 20 μl yeast suspension (5 × 10^7^ cells/ml in PBS) and 20 μl NBT (1.5 mg/ml in PBS) was added to every single well. The Wells that received 20 μl PBS and 20 μl DMSO were used as controls. The cells were later incubated for one hour at 37°C, the supernatant was then discarded and the adherent macrophages were washed with RPMI 1640 three times. The cells were air-dried before the 120 μl of 2M KOH and 140 μl DMSO were added to each well. The absorbance of the blue solution was measured at 570 nm (OD 570) in the plate reader. The percentage of NBT reduction (which reflects phagocytic activity) was calculated according to the following equation (32).


Phagocytic⁢index=(ODsample-ODcontrol)/ODcontrol×100

### *In vitro* Pinocytosis Assay

This experiment was conducted to assess the impact of the Lemon and Ginger combination water extract on the innate immunity expressed by the pinocytic activity of macrophages. The effect of the extract on macrophage function was measured using the neutral red uptake method. The peritoneal mice macrophages were collected and cultured for 48 h with decreasing concentrations of herbal water extract (12.5–1.56 mg/ml) in a 96-well plate. One hundred μl of neutral red solution (7.5 mg/ml in PBS) was added to each well; then, the wells were incubated for 2 h. The supernatant was discharged and the cells in the 96-well plate were washed with PBS two times to remove the neutral red that was not brought into the macrophage. Then, 100 μl of cell lysis solution (ethanol and 0.01% acetic acid at the ratio of 1:1) were added to each well for the cells to lyse. After the incubation of the cells at room temperature overnight, the optical density was measured at 540 nm. The pinocytic activity was shown in terms of absolute OD values (reflecting dye uptake) (32).

### Gas Chromatography Coupled to Tandem Mass Spectrometry Analysis

To identify the compounds in the extract, a chromatograph 2010 was used (ultra; Shimadzu, Tokyo, Japan). This is equipped with an 8030 mass detector, and the process is easily implemented with the aid of MS Lab Solution software. The cleaning process of the glass apparatus is thorough and involves applying soap with warm water three times, before being heated at 105°C for 1 h, and then cooled to 25°C. Sample bottles and equipment were cleaned with acetone and DMSO before the start of the experiment. The extract was shaken and mixed using the ultrasound path for 6 min, then it was filtered using glass wool. After drawing the extract into small vials, 1 μl was injected into the GC-MS. The chromatographic separation was achieved using a capillary Rtx-5MS column (30 m × 0.25 mm i.d. × 0.25 μm film thickness, Restek, Bellefonte, PA, United States). The stationary phase of the column is composed of 5:95 diphenyl:dimethylpolysiloxane blend. The operating GC conditions were kept at 60°C for 5 min and set to reach 240°C at the rate of 3°C per min. The sample was injected at the injection temperature of 250°C and the injection volume was 1.0 μl in the 1:30 split ratio. Furthermore, helium (at a flow rate of 1.0 ml/min) was used as a carrier gas. The MS was obtained with electron impact ionization (70 eV) at full scan mode (40–500 m/z). The ion source and the transfer-line temperature were maintained at 200°C. The MS was taken through a centroid scan of the mass ranging from 40 to 800 amu. The components were identified based on retention index, library mass search database (NIST and WILEY), and comparing with the mass spectral data (33).

### Statistical Analysis

Data analysis was performed by employing mean ± SEM. The statistical significance among the groups was measured using SPSS (Statistical Package for Social Sciences, Chicago, IL, United States) one-way ANOVA. A *P* value of <0.05 was considered significant. Furthermore, the IC_50_ values were calculated for the lemon and ginger combination water extract in the different cell lines using non-linear regression in SPSS (version 25).

## Results

### Gas Chromatography Coupled to Tandem Mass Spectrometry Analysis

Alpha-pinene, alpha-terpineol, and terpinen-4-ol compose the essential components in the lemon and ginger combination water extract. The analysis of the lemon and ginger combination water extract, using GC-MS/MS, showed the presence of different compounds.

The lemon and ginger combination extract; contain a high concentration of alpha-pinene, alpha terpineol, and terpinen-4-ol with percentages of 11.5, 7.5, and 5.4%, respectively (Table 1). While geraniol, geranial, neral, δ-elemene, camphene, and borneol have a percentage value of 3.75, 3.02, 4.4, 4.06, 3.35, and 4.1%, respectively. Minor detected compounds are citronellol (1.6%), sabinene (2.26%), myrtonol (2.85%), and beta-pinene (2.9%).

**TABLE 1:** Major compounds identified in the lemon and ginger combination extract using GC-MS/MS method.

No	Compound	Formula	M.W	R.T	%
1	2-Heptanol	C_7_H_16_O	116.2	13.02	1.88
2	Tricyclene	C_10_H_16_	136	15.53	1.66
3	α-Pinene	C_10_H_16_	136	17.22	11.5
4	Camphene	C_10_H_16_	136	18.19	3.35
5	Sabinene	C_10_H_16_	136	18.54	2.26
6	B -Pinene	C_10_H_16_	136	19.25	2.9
7	Methylheptenone	C_8_H_14_O	126	20.54	1.6
8	o-Cymol	C_10_H_14_	134	21.8	1.1
9	Terpinolene	C_10_H_16_	136	22.0	0.75
10	Linalool	C_10_H_18_O	154	22.4	0.31
11	*Trans-*2-Pinanol	C_10_H_18_O	154	26.21	0.58
12	Camphore	C_10_H_16_O	152	26.4	0.80
13	Borneol	C_10_H_18_O	154	28.9	4.1
14	Terpinen-4-ol	C_10_H_18_O	154	29.9	5.4
15	Isogeranial	C_10_H_16_O	152	30.05	8.02
16	Cryptone	C_9_H_14_O	138	30.8	5.21
17	α-Terpineol	C_10_H_18_O	154	31.6	7.5
18	Myrtenol	C_10_H_16_O	152	32.0	2.85
19	Citronellol	C_10_H_20_O	156	32.4	1.6
20	Neral	C_10_H_16_O	152	32.8	4.4
21	Geraniol	C_10_H_18_O	154	33.2	3.75
22	Geranial	C_10_H_16_O	152	33.82	3.02
23	Bornyl acetate	C_12_H_20_O_2_	196	33.97	1.51
24	δ-elemene	C_15_H_24_	204	34.40	4.06
25	Citronellyl acetate	C_12_H_22_O_2_	198	35.01	3.95
26	α-Cubene	C_8_H_6_	102	35.45	2.11
27	Aryl-curcumene	C_15_H_22_	202	35.48	1.02
28	α-Funebrene	C_15_H_24_	204	36.02	5.8
29	Cubenol	C_15_H_26_O	222	36.48	2.22
30	Epiglobulol	C_15_H_26_O	222	37.44	1.5
31	Viridifloral	C_15_H_24_O	220	37.89	0.73
32	Limonene	C_12_H_10_	154.21	37.99	0.41

*GC-MS/MS, gas chromatography coupled to the tandem mass spectrometry; MW, molecular weight, RT, retention time.*

### Compounds Found in Lemon and Ginger Behave in a Synergistic Manner to Inhibit Different Cancer Cell Lines *in vitro*

Inhibition of cell proliferation, in correlation to dosage, was observed after the treatment of six cell lines with serial dilutions of water extract (25–0.195 mg/ml).

**FIGURE 1:**
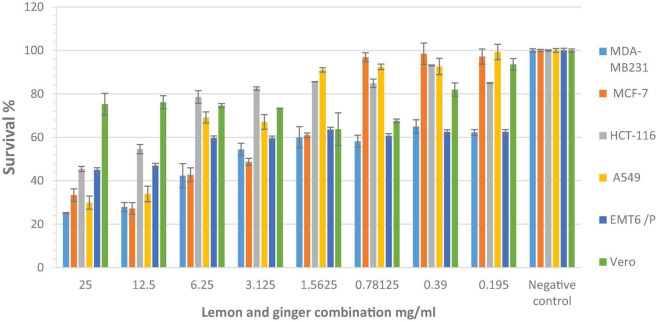
The antiproliferative activity of different concentrations (25–0.195 mg/ml) of the lemon and ginger water extract seen after the treatment of six cells with serial dilutions of water extract. *Significant values when compared with Vero cells proliferation (*P*-value = 0.05).

The lemon and ginger combination, at the highest concentration 25mg/ml, significantly (*P* value = 0.002) lowered the proliferation of MCF-7, MDA-MB-231, HCT-116, A549, EMT-6/P cell lines when compared with VERO normal cell line (Figure 1).

The IC_50_ values of lemon and ginger combination for MCF-7, MDA-MB-231, HCT-116, A549, EMT-6/P cell lines, and VERO normal cell line were 4, 3.5, 14, 6.5, 11, and >25 mg/ml, respectively (Figure 1). IC_50_ values were 3.5–11 mg/ml (Table 2).

**TABLE 2:** The IC_50_ values (mg/ml) for lemon and ginger extract and vincristine against various cell lines.

Analyzed plants	MCF-7 cell line	MDA-MB231 cell line	HCT-116 cell line	A549 cell line	EMT-6/P cell line	Vero-normal cells
Lemon and ginger IC_50_ (mg/ml)	4 ± 0.600	3.5 ± 0.500	14 ± 1.300	6.5 ± 2.000	11 ± 1.700	>25
Vincristine (positive control)	0.013 ± 0.008	0.025 ± 0.002	0.021 ± 0.009	0.008 ± 0.001	0.041 ± 0.006	>0.05

*Ten mice were used in each group.*

*mm^3^, cubic millimeter.*

### Lemon and Ginger Water Extract Decreased Vascular Endothelial Growth Factor Expression (*in vitro*) in Cancer Cells

Vascular endothelial growth factor (VEGF) was measured in the MDA-MB231 cell line to investigate whether the inhibition of angiogenesis may improve the antiproliferative effect. In the negative control group, VEGF returned a high outcome of (336 pg/ml).

**FIGURE 2:**
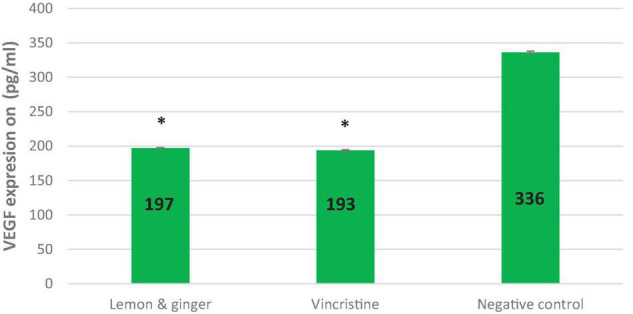
The effect of lemon and ginger treatment on the expression of vascular endothelial growth factor (VEGF). The concentration of VEGF (pg/ml) in the MDA-MB231 cell line treated with lemon and ginger combination (3.5 mg/ml) and vincristine (0.025 mg/ml), and in untreated control cells (negative control). Each treatment was performed in triplicated. Results are shown as means (bars) SEM (lines). *Significant values.

The lemon and ginger combination, concentrated at 3.5 mg/ml, significantly (*P* value = 0.02) lowered the level of VEGF expression (197 pg/ml). Vincristine lowered the value of VEGF to 193 pg/ml (*P* value = 0.01) (Figure 2).

### Lemon and Ginger Water Extract Induced Apoptosis by Enhancing Caspase-3 Activity

**FIGURE 3:**
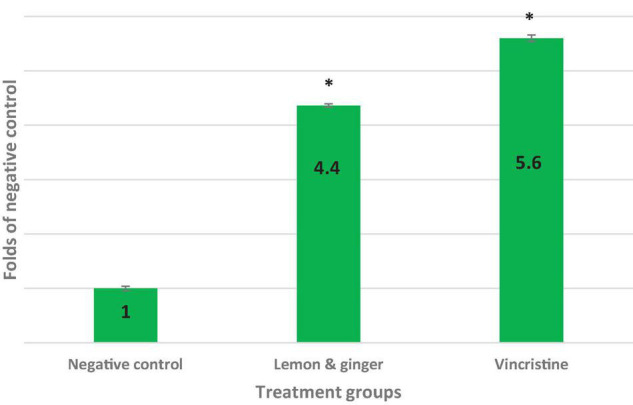
Folds of caspase-3 activity and apoptosis induction in IC_50_ concentrations of vincristine (0.025 mg/ml), lemon, and ginger combination (3.5 mg/ml) in MDA-MB231 cell line. *Significant values (*P*-value = 0.05).

The colorimetric assay of caspase-3 activity was conducted using the caspase-3 assay kit to estimate the impact of lemon and ginger combination water extract on the caspase-3 levels of the MDA-MB231 cell line. At the concentration of 3.5 mg/ml, the combination extract showed a value that was 4.4 times significantly (*P* value = 0.003) greater than that of the negative control. In contrast, vincristine showed a value equivalent to 5.6-folds (*P* value = 0.001) of the negative control (Figure 3).

### Lemon and Ginger Water Extract Enhances Lymphocyte-Proliferation in the Presence and Absence of Mitogens

**FIGURE 4:**
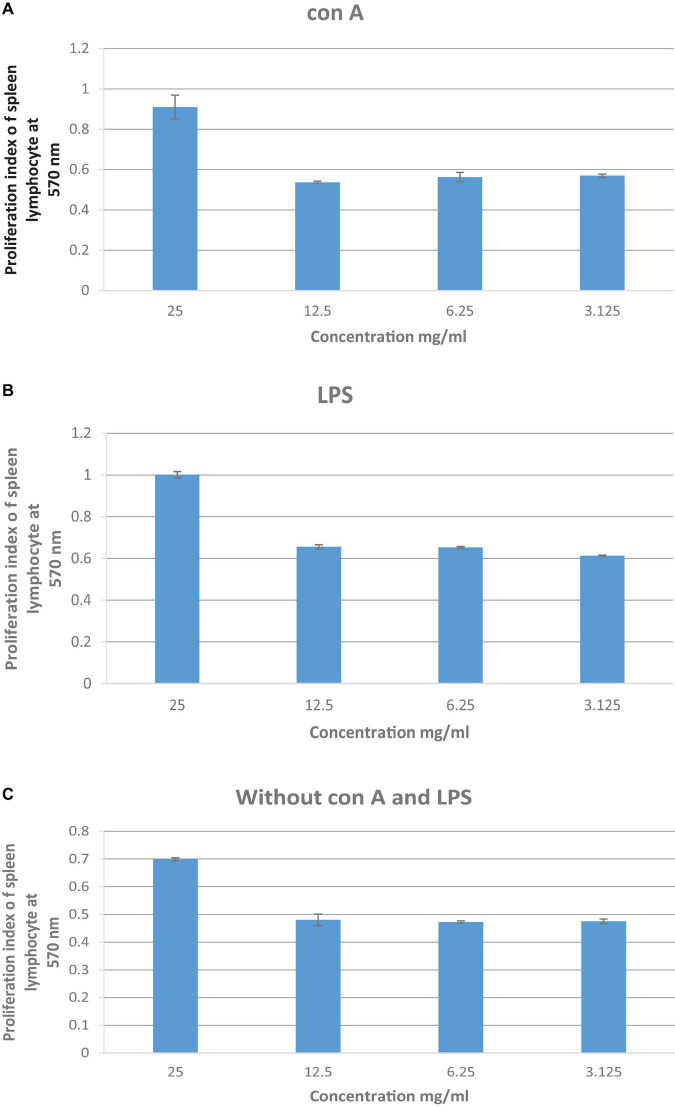
**(A)** The effect of lemon and ginger combination water extract at various concentrations (25–3.125 mg/ml) on the proliferation index of splenic lymphocytes in the presence of Con A (2 μg/ml). **(B)** The effect of lemon and ginger combination water extract at various concentrations (25–3.125 mg/ml) on the proliferation index of splenic lymphocytes in the presence of LPS (4 μg/ml). **(C)** The effect of lemon and ginger combination water extract at various concentrations (25–3.125 mg/ml) on the proliferation index of splenic lymphocytes in the absence of mitogens. Results are expressed as means of three independent experiments (bars) ± SEM (lines). Proliferation index = treated cell absorbance/negative control absorbance.

The combination of lemon and ginger induced lymphocytes cell proliferation in the presence of Con A and LPS (Figure 4). At the high concentration (25 mg/ml), the lemon and ginger combination water extract did not stimulate the proliferation of lymphocytes, with a cell viability index of 0.9 and 1 compared to the negative control on Con A and LPS stimulated cells, respectively. The viability cell index of the lemon and ginger combination water extract was 0.7 in the condition of no mitogenic effect (Figure 4).

### The Effect of Lemon and Ginger Combination Water Extract on Stimulating of Peritoneal Macrophages

**FIGURE 5:**
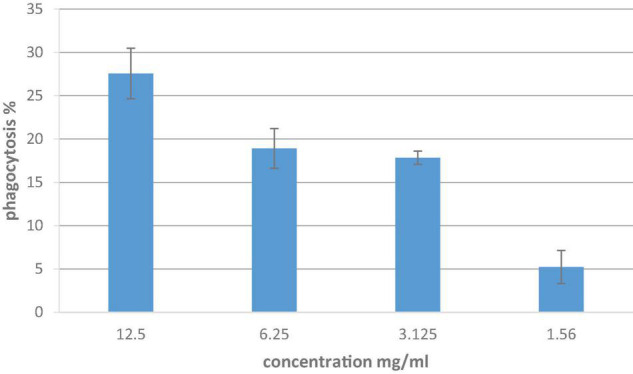
*In vitro* phagocytic assay utilizing nitro blue tetrazolium (NBT) reduction test of peritoneal macrophage treated with different concentrations of lemon and ginger combination water extract for 48 h. Results are expressed as means of three independent experiments (bars) ± SEM (lines).

**FIGURE 6:**
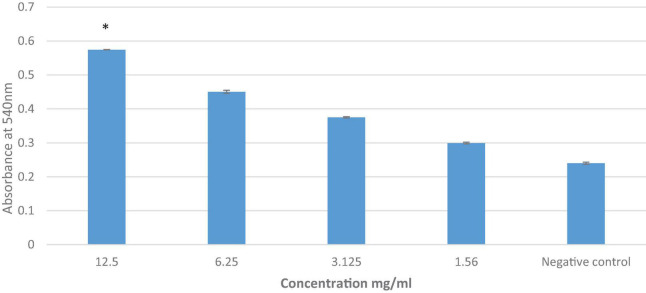
The effect of lemon and ginger combination water extract on macrophage pinocytosis. Results are expressed as means of three independent experiments (bars) ± SEM (lines). *Significant effect of the herbal drink on the pinocytosis activity at the highest concentration when compared with negative control.

The lemon and ginger combination water extract showed no phagocytic activity with a percentage value of 27.6% at different doses ranging from 12.5 to 1.56 mg/ml (Figure 5). While the results of the pinocytic assay revealed that the lemon and ginger combination water extract caused an increase in pinocytic activity (*P* = 0.004) with an absorbance value of 0.57 nm compared to the control absorbance value of 0.24, at different doses ranging from 12.5 to 1.56 mg/ml (Figure 6).

### The Prevention and Curative Effects of Lemon and Ginger Combination Water Extract Against EMT-6/P Cells Implanted in Mice

A significant decrease in tumor size was noticed in tumor-bearing mice treated orally with the lemon and ginger combination compared with the negative control that showed tumor growth increased by (107.0197%) (Table 3).

**TABLE 3:** Effect of lemon and ginger combination water extract on tumor size and cure percentage.

Treatment	Initial tumor size mm^3^	Final tumor size mm^3^	% change in tumor size	% of mice with no detectable tumor	Average tumor weight (g)
Control	432.6216 ± 22.3	895.6118 ± 43.1	107.0197	20%	0.715
Lemon and ginger combination	242.5815 ± 19.7	162.9365 ± 20.5	-32.8323	50%	0.288

**FIGURE 7:**
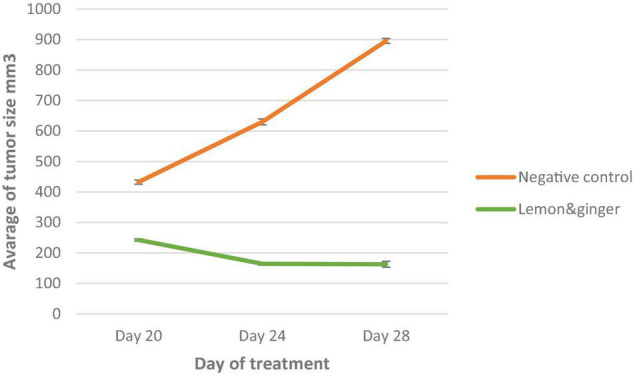
A plot of change in average tumor size (mm^3^) vs. time in (days of treatment in EMT-6/P cell line). *Significant values (*P*-value = 0.05).

**FIGURE 8:**
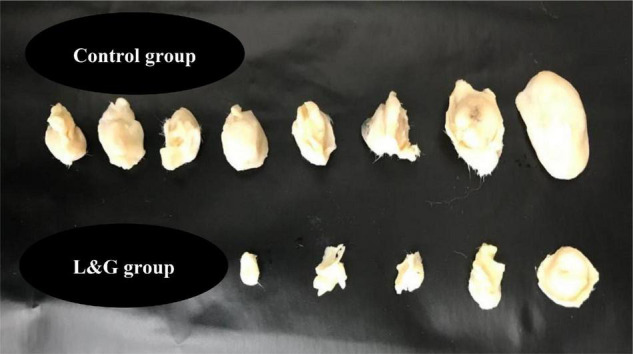
The effect of lemon and ginger combination water extract (L & G) on tumor size and cure percentage. Combination therapy resulted in a high cure percentage and small tumors size. There were 10 mice in each group.

The lemon and ginger combination recorded a percentage in tumor reduction of (32.8%) with no recorded deaths and a percentage of mice with no detectable tumor of (50%) (Figures 7, 8). On the other hand, treated mice showed normal activity with no side effects.

## Discussion

Herbs are viewed to be of high value to the health of individuals and communities in general. The medicinal benefit of plants lies in some chemical substances which have specific physiological effects on the human body (33). The water extract of lemon and ginger combination showed potent antiproliferative activity against the MCF-7, MDA-MB231, HCT-116, A549, EMT-6/P, and Vero (normal cells) cell lines (Table 1).

No previous studies have examined the antiproliferation activity of the lemon and ginger combination water extract on the aforementioned cell lines. However, many studies have investigated the antiproliferation activity of each extract against selected cell lines.

Lemon has shown the presence of different indolofuroquinoxaline derivatives, which exhibited antiproliferative activity against leukemia and human breast cancer cells. On the other hand, there was no significant activity against the normal cell line (34).

Meanwhile, ginger has been reported to have a cytotoxic effect against human breast cancer cell lines (MDA-MB-231). The ability of ginger to inhibit cell growth was due to the presence of [6]-gingerol as it hindered the activity of matrix metalloproteinases (MMP)-2 and MMP-9 in the human breast cancer cells (35).

The lemon and ginger combination water extract revealed synergic effect in reducing cancer cells proliferation *in vitro*. This finding is compatible with a previous study, which reported the ability of lemon and garlic water extract to induce apoptosis and prevent carcinoma development (BR35A,). However, the potent anticancer response of the lemon and ginger combination water extract was observed to be mediated by diverse anticancer mechanisms, which may be explained by the existence of various phytochemicals compounds. The observed proliferation inhibition in the cells treated with the lemon and ginger combination water extract is basically because of the impact of different antiproliferation factors. Alpha-pinene was detected in lemon and ginger combination utilizing GC/MS with a percentage value of 11.5%.

The cytotoxic potential of alpha-pinene was examined in two different human cell lines (human ovarian cancer cell line SK-OV-3 and human hepatocellular carcinoma cells Bel-7402) (37). Also, it was studied against the human histiocytic lymphoma U937 cell line (38). Furthermore, a previous study confirmed that alpha-pinene has an antiproliferation effect through provoking oxidative stress and associated signaling cascade against human lung cancer (A549) and human liver cancer (HepG2) (39). These previous findings are consistent with this study. On the other hand, α-pinene was found to have no antiproliferation activity against melanoma cells *in vitro* (40). This may be justified by the fact that solid tumors have different responses to phytochemicals as α-pinene.

Other highly concentrated compounds in the combination extract are alpha-terpineol (7.5%) and terpinen-4-ol (5.4%).

Alpha-terpineol has demonstrated antioxidant ability against peroxyl radicals and has been shown to have a cytostatic impact against six human cancerous cell lines from five diverse histologic and embryonic origins (breast, lung, prostate, ovarian, and leukemia) (41). Furthermore, terpinen-4-ol was found in the combination extract to a significant degree. It caused necrotic cell death coupled with less activity of apoptosis process in both murine malignant mesothelioma cell line AE17 and murine B16 melanoma cells. The inhibitory impact of terpinen-4-ol is achieved through the elicitation of the G1 cell cycle arrest (42).

Further analysis of the lemon and ginger combination water extract revealed the presence of geraniol, citral, δ-elemene, camphene, and *borneol* in notable percentages and the previously reported activity of these components confirms the results of this study.

Based on a previous study, the growth of prostate cancer cells was inhibited by the effect of geraniol compound on inducing the apoptosis and autophagy process (43). Citral prevents the proliferation of breast cancer cells (MDA MB-231 cells) *in vitro*, mainly through the initiation of apoptosis, antimetastasis, and antiangiogenesis potentials (44). In colorectal adenocarcinoma (DLD-1), the antiproliferative activity of δ-elemene was implemented by the activation of mitochondrial caspase-dependent and caspase-independent pathways (45). On the other hand, camphene exhibited anticancer activity in melanoma cells both *in vitro* and *in vivo*. It can disrupt the mitochondrial membrane potential and enhances the caspase-3 effect (46). In a previous study, borneol (NB) improved the absorption of selenocysteine; as a result, it increased his apoptotic activity against human hepatocellular carcinoma cells. Also, they reduced tumor growth by provoking ROS-mediated DNA damage (47).

In the current study, the potential inhibition of cancer cell growth by lemon and ginger combination may due to the effects of the nine major compounds previously mentioned and to the other active minor components.

To gain further in-depth knowledge of the mechanisms of action of the combination extract under study, the lemon and ginger combination was further tested for its ability to inhibit angiogenesis. The VEGF is a potent signal protein that stimulates angiogenesis. VEGF upregulation is a well-known mechanism in many types of tumors and the inhibition of this pathway is an interesting target in cancer prevention and therapy (48). In our study, the lemon and ginger combination showed a strong ability to suppress the MDA-MB-231 human breast cancer angiogenesis.

Further examination showed that the lemon and ginger combination water extract had a potent anticancer effect *in vitro*. The combination has potent apoptosis induction activity and potent VEGF inhibition in the MDA-MB231 cell line.

In MDA-MB-231 cells, nuclear factor kappa B (NF-κB) is involved in the upregulation of VEGF mRNA then increases the angiogenesis (49). The activations of NF-κB were attenuated utilizing α-pinene treatment (50). In HeLa cells, alpha-terpineol repressed TNF-α and NF-kB translocation into the cell nucleus. Repression of NF-kB activation can result in the decrease of cyclin D expression, an essential protein in the induction of the G phase of the cell cycle (51). Meanwhile, geraniol is a strong agent with antiangiogenic properties (52). These results are inconsistent with the findings of this study.

Another mechanism responsible for the observed anticancer activity is apoptosis (programmed cell death) induction. In cancer, this process is inoperative, due to the upregulation of antiapoptotic genes and the downregulation of apoptotic genes; therefore, cells continue dividing and proliferating (53). One of the targeted pathways in cancer treatment is the activation of apoptosis.

In our study, the lemon and ginger combination herbal drink was found to have the ability to induce apoptosis in the MDA-MB231 cell line (4.4 times greater than that of the negative control).

Both hesperidin (flavonoid) (54), and eriocitrin (flavonoid) (55) in lemon cause apoptosis induction and inhibit the human hepatocellular carcinoma. The high antiproliferative activity of lemon has been explained in previous studies, which have shown that the methanol extract of lemon has anticancer activity toward MCF-7 breast cancer cells *via* Bax-related caspase-3 activation. Meanwhile, the ethanol extract of lemon peel exhibited a weak cytotoxic effect (IC_50_ > 500 μg/ml) against human leukemia HL-60 cells (56). Alpha-pinene, which is found in high percentages in the lemon and ginger combination, was also capable of inducing apoptosis, as evidenced by the rise in caspase-3 activity (57). Moreover, geraniol has the most dominant apoptosis-inducing activity among terpenoids against shoot primordia of *Matricaria chamomilla*; it causes DNA fragmentation in the cells. The activity of geraniol is concentration- and time-dependent (58). These results correspond with this study’s conclusion.

The immune system is a sophisticated defense system in vertebrates, having the role of protecting them from numerous types of foreign infectious agents that they encounter during their lifetimes. It uses a variety of cells, tissues, and organs and is capable of recognizing and eliminating invasions (59). There has been a growing interest in identifying and characterizing natural compounds with immunomodulatory activities. Three immune assays have been conducted, namely, lymphocytes proliferation, phagocytosis, and pinocytosis assays.

Lymphocytes proliferation assay was conducted three times: splenic lymphocytes with extract alone, splenic lymphocytes with extract and Con A, and splenic lymphocytes with extract and LPS, for lemon and ginger combination water extract.

### Lemon and Ginger Combination Water Extract Had a Limited Effect on Lymphocyte Proliferation and Phagocytosis Activity

The volatile oil of ginger exhibited significant suppression of the mitogen-stimulated T-lymphocyte proliferation in mice (60), which agrees with our result. While citrus peels had a mitogenic response to con A (56). In addition to that, both lemon citrus and ginger stimulate innate immunity (61, 62). These previous studies conflicted with our results may be due to the antagonistic effect of the combination on the activity of both lemon and ginger.

For pinocytic activity, the potent pinocytic activity was the lemon and ginger combination. Various groups of phytochemicals are found in the lemon and ginger combination water extract. This could explain the activation of a specific anticancer response, such as immune enhancement It is worth mentioning that *a*-pinene exhibited anti-Leishmania activity *via* macrophage stimulation with minimum cytotoxicity (63). Citral influences cytokines production by activation of murine macrophages through the prevention of the transcription factor NF-κB (64). In the cell-based assay, terpinen-4-ol was able to modulate apoptosis and immune system activity in a way that reduced the cell growth of melanoma cells (65). In agreement with these previous studies, the results of this study showed that the lemon and ginger extract possesses strong antiproliferation and pinocytosis activity.

An *in vivo* study was conducted to evaluate the activity of lemon and ginger combination water extract on the EMT-6/P cell line implanted in Balb/C mice. This study results showed a significant reduction in tumor sizes in all treated mice. On the other hand, no previous study has examined the *in vivo* tumor-prevention activity of lemon and ginger combination water extract.

Besides cancer chemotherapy strategies, using dietary agents such as fruits and vegetables help the prevention of tumor growth. Extracts derived from fruits and vegetables are demonstrated to have antiproliferative effects (18). It is extensively acknowledged that chemopreventive agents, as lifestyle and dietary habits, have more superior potential in the long term than chemotherapeutic agents, especially in prostate cancer growth and progression (65).

*In vivo*, the lemon and ginger combination water extract induced a significant decrease in percentage change in tumor size, a high percentage of mice with no detectable tumor, and low average tumor weight (g) (Table 2).

A cohort study confirms that the daily consumption of citrus fruits is associated with a decreased risk of gastric cancer (18). Notably, daily oral feeding of 100 mg/kg of whole ginger water extract prevented both the growth and progression of the human prostate cancer cell line PC-3 xenografts by approximately 56% in nude mice (66). The lemon and ginger combination is mostly composed of alpha-pinene, according to GC-MS/MS analysis. *In vivo*, alpha-pinene pretreatment reduced the production of the pancreatic tumor necrosis factor, interleukin (IL)-1beta, and IL-6 during the induction of acute pancreatitis by cerulean (67). Another previous study found that the tumor volumes from mice treated with α-pinene were around 40% less than those from the mice in the control group (68). There is an interaction between the work of lemon and ginger combination resulted in antitumor activity *in vitro* and *in vivo*. Furthermore, the anticancer activity could be explained by the stimulation of apoptosis and pinocytosis and reduction of VEGF expression.

## Conclusion

Lemon and ginger herbal combination is a healthy drink with anticancer and immunomodulatory health benefits. Its anticancer effect is mediated by apoptosis induction and angiogenesis inhibition. The immunomodulatory effect is mediated through the activation of the innate immune system. The health benefits of this herbal drink are mainly due to the presence of biologically active phytochemicals in ginger and lemon. Further studies are needed to have a better understanding of the mechanisms of action of the herbal drink.

## Data Availability Statement

The raw data supporting the conclusions of this article will be made available by the authors, without undue reservation.

## Ethics Statement

The animal study was reviewed and approved by Institutional Research Board at Applied Science Private University.

## Author Contributions

The project idea was developed by WT. IA-A performed the sample collections and ran the laboratory experiments. Both authors developed the experimental design, analyzed the data, and wrote and revised the manuscript.

## Conflict of Interest

The authors declare that the research was conducted in the absence of any commercial or financial relationships that could be construed as a potential conflict of interest.

## Publisher’s Note

All claims expressed in this article are solely those of the authors and do not necessarily represent those of their affiliated organizations, or those of the publisher, the editors and the reviewers. Any product that may be evaluated in this article, or claim that may be made by its manufacturer, is not guaranteed or endorsed by the publisher.
